# AM6527, a neutral CB1 receptor antagonist, suppresses opioid taking and seeking, as well as cocaine seeking in rodents without aversive effects

**DOI:** 10.1038/s41386-024-01861-y

**Published:** 2024-04-10

**Authors:** Omar Soler-Cedeño, Hannah Alton, Guo-Hua Bi, Emily Linz, Lipin Ji, Alexandros Makriyannis, Zheng-Xiong Xi

**Affiliations:** 1 Addiction Biology Unit, Molecular Targets and Medication Discovery Branch, Intramural Research Program, National Institute on Drug Abuse, Baltimore, MD USA; 2 Postdoctoral Research Associate Training (PRAT) Fellow, National Institute of General Medical Sciences, Bethesda, MD USA; 3 Center for Drug Discovery, Department of Pharmaceutical Sciences, Northeastern University, Boston, MA USA; 4 Department of Chemistry and Chemical Biology, Northeastern University, Boston, MA USA

**Keywords:** Addiction, Pharmacology

## Abstract

Preclinical research has demonstrated the efficacy of CB1 receptor (CB1R) antagonists in reducing drug-taking behavior. However, clinical trials with rimonabant, a CB1R antagonist with inverse agonist profile, failed due to severe adverse effects, such as depression and suicidality. As a result, efforts have shifted towards developing novel neutral CB1R antagonists without an inverse agonist profile for treating substance use disorders. Here, we assessed AM6527, a CB1R neutral antagonist, in addiction animal models. Our findings revealed that AM6527 did not affect cocaine self-administration under fixed-ratio reinforcement schedules but dose-dependently inhibited it under progressive-ratio reinforcement schedules. Additionally, AM6527 dose-dependently inhibited heroin self-administration under both fixed-ratio and progressive-ratio reinforcement schedules and oral sucrose self-administration under a fixed-ratio reinforcement schedule, as well as cocaine- or heroin-triggered reinstatement of drug-seeking behavior in rats. However, chronic AM6527 administration for five consecutive days significantly inhibited heroin self-administration only during the initial two days, indicating tolerance development. Notably, AM6527 did not produce rewarding or aversive effects by itself in classical electrical intracranial self-stimulation and conditioned place preference tests. However, in optical intracranial self-stimulation (oICSS) maintained by optogenetic stimulation of midbrain dopamine neurons in DAT-cre mice, both AM6527 and rimonabant dose-dependently inhibited dopamine-dependent oICSS behavior. Together, these findings suggest that AM6527 effectively reduces drug-taking and seeking behaviors without rimonabant-like adverse effects. Thus, AM6527 warrants further investigation as a potential pharmacotherapy for opioid and cocaine use disorders.

## Introduction

Substance use disorders (SUD), characterized by persistent drug use despite serious negative consequences, constitute a severe health and social problem worldwide. In the United States, costs related to SUD exceed $740 billion per year [1], and in 2021 drug overdose deaths reached the alarming total of 100,000 deaths annually [2]. Although several medications have been approved for the treatment of opioid or nicotine use disorders [3–8], the relapse rate to opioids or nicotine remains high. Furthermore, there is no current approved medication for the treatment of psychostimulant (such as cocaine, methamphetamine) use disorders [9, 10]. Therefore, the search for effective treatments for SUD is a priority in medication discovery research.

The cannabinoid CB1 receptor (CB1R) has received much attention in medication development for the treatment of SUD in the past decades due to its high expression in the brain reward system [11–15] and its functional involvement in addiction-related behavior [16–18]. Rimonabant is a selective CB1R antagonist with an inverse agonist profile that clinically emerged as a treatment for obesity in Europe and preclinically showed effectiveness for treating SUD [19–22]. However, the clinical trials with rimonabant for the treatment of obesity failed due to the severe side effects such as nausea, emesis, depression, and suicidal tendencies, which led to its discontinuation from clinical trials [22–24] (see comprehensive reviews by [22, 25]). The mechanisms underlying rimonabant’s adverse psychiatric effects are not well understood but have been proposed to be related to its inverse agonist actions. Therefore, research efforts have shifted to develop CB1R neutral antagonists without an inverse agonist profile [19, 26], leading to the development of a series of novel compounds such as AM4113, PIMSR, AM6527, AM6545, and ABD459 [26–28].

Several of these neutral CB1R antagonists have been tested in experimental animals for their therapeutic effects against SUDs with mixed results. For example, AM4113 inhibits drug (heroin, nicotine) self-administration and reinstatement of drug-seeking behavior in rats and non-human primates [29–33], while AM4113 itself lacks many of rimonabant-like side-effects such as nausea, vomiting, anxiety, and aversion [32, 34–36]. These findings suggest that AM4113 has an improved safety profile over rimonabant. However, a pharmacokinetic study indicates that AM4113 has poor oral bioavailability [29]. AM4113 is also reported to produce anxiety-like effects similar to that by rimonabant in an open field locomotion test [34], which may limit its development as a clinical treatment [37]. PIMSR is another neutral CB1R antagonist with a similar binding affinity for human CB1R (to rimonabant [38]). We recently reported that PIMSR inhibits cocaine self-administration, reduces cue-induced reinstatement of cocaine seeking, and decreases cocaine-enhanced brain-stimulation reward without rewarding or aversive effects by itself [39]. However, pharmacodynamic assays indicate that PIMSR displays relatively low brain penetration, suggesting a possible limitation as a treatment for SUDs [40].

In this study, we investigated another CB1R neutral antagonist, AM6527, in experimental animals. In contrast to AM4113, AM6527 is an orally effective CB1R neutral antagonist with ~100-fold selectivity for CB1R (Ki = 4.88 nM) over CB2Rs (Ki = 463 nM) [37]. It was reported that AM6527 inhibited food self-administration under FR5 reinforcement at 1-8 mg/kg after intraperitoneal administration or 4–16 mg/kg after oral administration [37]. AM6527, at 5 mg/kg orally, is also effective in preventing naloxone-precipitated opioid withdrawal [41]. However, little is known about whether systemic administration of AM6527 can inhibit cocaine or heroin self-administration and reinstatement of drug-seeking behavior with or without rimonabant-like side-effects. Here, we report that AM6527 is effective in attenuating cocaine or heroin addiction-related behavior in rats and optical brain-stimulation reward in mice, while AM6527 by itself did not produce rewarding or aversive effects in electrical brain-stimulation reward and CPP models, suggesting that AM6527 could act as a potential therapy for cocaine or opioid use disorders.

## Material and methods

### Animals

All experimental procedures were approved by the National Institute on Drug Abuse Animal Care and Use Committee. The subjects included in this study consisted of 84 male Long-Evans rats (purchased from Charles River Laboratories, Frederick, MD) and 11 male and female heterozygous DAT-cre mice (breeders purchased from Jackson Laboratory, Bar Harbor, ME; B6.SJL-Slc6a3tm1.1(Cre)Bkmn/J; stock # 006660), aged 8–24 weeks. Animals were housed in climate-controlled animal colony rooms on a 12-h reversed light-dark cycle (lights on at 7:00 p.m., lights off at 7:00 a.m.) with free access to food and water throughout the study. The housing conditions and animal care were consistent with the Guide for the Care and Use of Laboratory Animals (National Research Council, 2011).

### Drugs

Heroin and cocaine hydrochloride were obtained through the NIDA Pharmacy. AM6527 and rimonabant were obtained from the Center of Drug Discovery at Northeastern University, Boston, MA, and were dissolved in 5% cremophor EL (Kolliphor EL, MilliporeSigMa).

### Experiment 1: Intravenous cocaine or heroin self-administration in rats

#### Surgery

Right jugular vein intravenous (i.v.) catheterization surgery and i.v. self-administration procedures were conducted in Long-Evans rats as previously described [32, 42]. Briefly, animals were anaesthetized with ketamine/xylazine (100/10 mg/kg), and a catheter, constructed of micro-renathane (Braintree Scientific Inc., Braintree, MA, USA), was inserted into the right jugular vein. After being sutured into place, the catheter was passed subcutaneously to the top of the skull and mounted onto the skull. To prevent clogging, the catheters were flushed daily with a gentamicin-heparin-saline solution (30 IU/ml heparin) (ICN Biochemicals, Cleveland, OH, United States).

#### Self-administration under an fixed-ratio (FR) reinforcement schedule

After recovery from surgery, animals were placed in the self-administration chambers (Model MED-008-CT-B1) and trained to self-administer cocaine (0.5 mg/kg/infusion) or heroin (0.05 mg/kg/infusion) daily under fixed-ratio (FR1 in the first week, FR2 in the following training sessions) schedules. Each training session lasts 3 h or ends immediately when the maximum of 50 infusions has been reached. Animals were trained until their behavioral performance reached stable cocaine or heroin self-administration levels, defined as ≥20 cocaine or ≥15 heroin infusions per 3 h session; <20% variability in daily cocaine or heroin infusions across two consecutive sessions; and an active/inactive lever pressing ratio exceeding 2:1. Then, we evaluated the effects of AM6527 (10, 30 mg/kg, i.p.) or vehicle (5% cremophor equivalent volume) on cocaine or heroin self-administration under an FR2 reinforcement schedule in rats. A within-subjects design was used in this experiment. After each test, the animals continued daily cocaine or heroin self-administration until a stable baseline was reestablished. The order of drug doses was counterbalanced. The time intervals between different drug/dose tests were 3–5 days.

#### Self-administration under a progressive ratio (PR) reinforcement schedule

In rats, drug self-administration training under an fixed-ratio schedule was conducted until stable levels of behavioral performance were obtained. Then, cocaine or heroin self-administration was continued under a progressive ratio (PR) reinforcement schedule following the protocols we have previously described [42]. Briefly, after stable self-administration under a FR2 schedule of reinforcement was established, rats were switched to drug self-administration under a PR schedule, during which the lever-pressing work requirement needed to receive a single i.v. drug infusion was progressively raised within each test session according to the following PR series: 1, 2, 4, 6, 9, 12, 15, 20, 25, 32, 40, 50, 62, 77, 95, 118, 145, 178, 219, 268, 328, 402, 492, and 603 until a break point was reached. The breakpoint was defined as the maximal workload (i.e., number of active lever presses) completed for the last drug infusion prior to a 1-h period during which no infusions were obtained by the animal. After a stable breakpoint was established, subjects were assigned to different subgroups to determine the effects of AM6527 (3, 10, 30 mg/kg, i.p.) or vehicle (30 min prior to test) on PR breakpoint for heroin or cocaine self-administration. Because it is difficult to re-establish a stable breakpoint level after each drug test, we used a between-subjects design to determine the dose–response effects of AM6527 on breakpoint for cocaine or heroin in rats.

#### Cocaine- and heroin-induced reinstatement of drug seeking

In this set of experiments, we assessed whether AM6527 reduces cocaine or heroin-induced reinstatement of drug-seeking behavior. Here six different groups of rats (*n* = 7–9 in each group) were trained to self-administer cocaine (0.5 mg/kg/infusion) or heroin (0.05 mg/kg/inf) under a FR2 schedule of reinforcement, as described in Exp. 1. After 2-3 weeks of self-administration, rats underwent extinction training during which responding on either lever produced no consequences. Once rats reached the extinction criterion (< 20 lever presses within their 3-h session), they were randomly divided into 3 dose groups to test the effects of AM6527 on cocaine- or heroin-induced reinstatement of drug seeking. Each group of rats received only one of the AM6527 doses (0, 10 or 30 mg/kg, i.p.), 30 min prior to the onset of the session, and then received cocaine (10 mg/kg, i.p.) or heroin (1 mg/kg, i.p.) priming 15 min before the beginning of the reinstatement test.

#### Effects of chronic administration of AM6527 on heroin self-administration

We also assessed the effects of repeated daily administration of AM6527 on heroin self-administration maintained by 0.05 mg/kg/infusion under an FR2 reinforcement schedule for 5 consecutive days. The chronic AM6527 dose was chosen based on our finding that AM6527, at 30 mg/kg, significantly inhibited heroin self-administration under both FR2 and PR reinforcement schedules. After stable self-administration was achieved, rats received either the vehicle (5% Cremophor) or 30 mg/kg of AM6527, respectively, 30 min before each of the daily heroin self-administration session. The animals then continued heroin self-administration for additional three sessions without AM6527 pretreatment. Animals’ responses on the levers and heroin infusions were recorded.

### Exp. 2: Oral sucrose self-administration in rats

Procedures for oral sucrose self-administration in rats were the same as we reported previously [43]. Briefly, rats were trained to self-administer sucrose under an FR1 schedule of reinforcement during daily 1-h sessions. Responding on the active lever activated the syringe pump causing the delivery of 5% liquid sucrose onto a liquid food receptacle (0.1 mL per delivery) and the presentation of the light/tone cue above the active lever. Responses on the inactive lever were counted but had no consequences. During the 4.2-s infusion period, additional responses on the active lever were recorded but did not lead to additional infusions. To prevent satiation of sucrose reward, we set a maximal number of 100 sucrose deliveries during each 1-h session. After stable sucrose self-administration was achieved, defined as (i) at least 20 sucrose rewards earned per 1-h session, (ii) less than 20% variability in daily sucrose intake across two consecutive sessions, and (iii) an active/inactive lever press ratio exceeding 2:1, the rats were randomly received vehicle treatment or one of two doses of AM6527 (3, 10 mg/kg, i.p.). The treatment was counterbalanced in each rat, and each test was separated by 3-5 additional training sessions. The total number of sucrose deliveries during the 1-h self-administration session and the numbers of active and inactive lever responses were used to evaluate the effects of AM6527 on oral sucrose self-administration.

### Experiment 3: Electrical intracranial self-stimulation (eICSS) in rats

The experimental procedures for electrical intracranial self-stimulation were performed as previously described [42]. Briefly, rats were surgically implanted with a unilateral monopolar stainless steel stimulating electrode (Plastics One, Roanoke, VA, USA) targeted at the medial forebrain bundle at the level of the lateral hypothalamus under 100/10 mg/kg ketamine/xylazine anesthesia. Rats were allowed a minimum of 7 days to recover from surgery prior to the start of eICSS experiments. After animals acquired stable eICSS behavior, we tested the effects of AM6527 (3, 10, 20 mg/kg, i.p.) on rewarding eICSS sehavior. We chose these drug doses that are relatively lower than those used in the above drug self-administration (3, 10, 30 mg/kg, i.p.) because AM6527, at 10 mg/kg, produced an inhibitory effect on Ymax in eICSS. Thus, 30 mg/kg AM6527 may produce profound motor effects, complicating the data interpretations. A within-subjects design was used to evaluate the effects of AM6527 on eICSS behavior with intervals between each dose tests 3–5 days.

### Experiment 4: Conditioned place preference in mice

The CPP procedures are the same as we reported previously [44]. Briefly, we used 2 days of preconditioning and 4 days of AM6527 conditioning, followed by two days of testing. On days 1–2, mice were placed in the center corridor and provided free access to the other two compartments for 15 min daily. The time spent in each compartment was recorded. This habituation was to eliminate biased mice (defined operationally as spending over 800 s in either compartment) [44]. On each of the next 7 days (days 3–9) of the conditioning phase, mice received vehicle (5% cremophor, i.p.) or one dose of AM6527 (30 mg/kg, i.p.), then saline (i.p.) on alternate days, after which each mouse was confined to the randomly designated treatment-appropriate compartment for 45 min. Drug-paired and saline-paired compartments were counterbalanced between groups. On days 10–11 of the CPP testing phase, the mice were again placed in the center corridor and provided free access to the other two compartments for 15 min. During this phase, no AM6527 or saline was given. The computer recorded the time spent in each chamber. The preference (i.e., CPP score) was assessed by time (sec) spent in the AM6527- or vehicle-paired compartment minus time spent in the saline-paired compartment. The average value of CPP scores pre- versus post-conditioning was used to evaluate the rewarding or aversive effects of AM6527 compared to vehicle control.

### Experiment 5: Optogenetic intracranial self-stimulation (oICSS) in DAT-Cre mice

The intracranial surgery and optical ICSS procedures used are the same as reported previously [14, 45]. Briefly, DAT-cre mice were anesthetized with a ketamine and xylazine mixture and placed in a stereotaxic device. Subjects received bilateral microinjections of an excitatory channelrhodopsin virus (AAV5-EF1α-DIO-ChR2-EYFP, University of North Carolina Gene Therapy Center) in the VTA (AP −3.28, ML ± 0.43, DV −4.41; 10° angle from the midline). Then, custom built ferrule fibers were implanted 0.5 mm above the virus injection sites and secured to the skull using dental cement [14]. After four weeks of recovery from the intracranial surgery, subjects were tested in operant chambers for oICSS. Initially, mice were placed on an FR1 schedule in which a single response on the active lever led to a 1-s pulse of laser stimulation (473 nm, 20 mW, 5 ms duration, 50 Hz) and a 1-s activation of the cue light above the active lever. Responses on the inactive lever had no programmed consequences. Daily sessions were 60 min in length. Once stable lever pressing was observed, a rate-frequency oICSS procedure was instituted. In this program, 6 stimulation frequencies (100, 50, 25, 10, 5, 1 Hz) were available in descending order during sessions, with a new frequency presented every 10 min.

After stable stimulation-response curve was achieved, mice received an i.p. injection of rimonabant (3, 10 mg/kg), or AM6527 (3, or 10 mg/kg) 15 min prior to the test session and allowed to lever press for oICSS. As the oICSS is maintained by direct stimulation of midbrain DA neurons, it is highly sensitive to a test drug that acts on the mesolimbic DA system directly or indirectly [14, 46, 47]. As functional CB1R has been identified in a subset of midbrain DA neurons [48], lower doses (3, 10 mg/kg, i.p.) of AM6527 were used in this experiment. After each test, mice received additional oICSS sessions until a new baseline was established. After completion of the above behavioral experiment, immunohistochemistry was used to verify AAV-ChR2-eYFP expression in VTA DA neurons in DAT-cre mice using the methods we reported previously [14, 48].

### Data analysis

All data are represented as the mean ± SEM. One-way (or repeated measures, RM) ANOVA were utilized to analyze effects of different AM6527 doses on drug self-administration and eICSS. Two-way ANOVA was utilized to analyze cocaine and heroin reinstatement tests. Two-way (RM) ANOVA was utilized to analyze AM6527 or rimonabant effects on oICSS and reinstatement responses. Paired *t*-tests were used to compare pre- versus post-conditioning AM6527 CPP scores and pre- versus post-conditioning vehicle CPP scores for the CPP experiment. Post hoc analyses were done using Holm-Sidak test compared to vehicle/baseline control group. The value of *p* < 0.05 was used as the minimally acceptable statistically significant difference value in all tests.

## Results

### AM6527 reduces cocaine self-administration under a PR, not FR2, reinforcement schedule

**Fig. 1:**
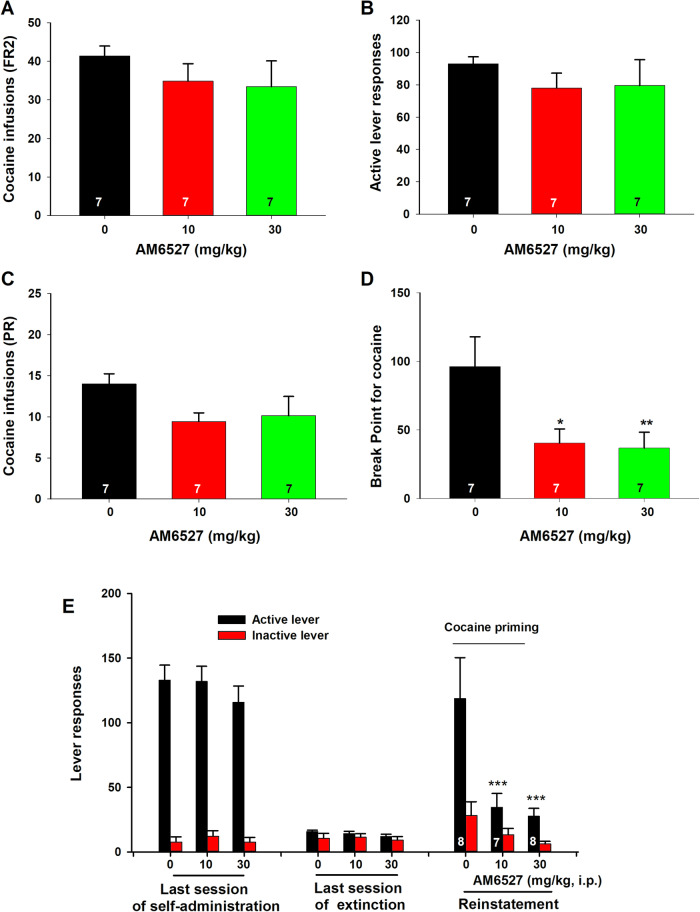
The effects of AM6527 on cocaine self-administration and reinstatement of cocaine-seeking behavior in rats. AM6527 failed to alter the total number of cocaine infusions **A** or active lever presses **B** under an FR2 schedule of reinforcement. AM6527 reduced cocaine self-administration under a PR schedule of reinforcement as assessed by the total number of cocaine infusions **C** and breakpoint for cocaine self-administration **D**. **E** AM6527 attenuated 10 mg/kg cocaine-triggered reinstatement of drug-seeking behavior. *p < 0.05; ** p < 0.01, ***p < 0.001, com*p*ared to the vehicle control group.

We first examined the effects of AM6527 on cocaine self-administration maintained by cocaine (0.5 mg/kg/infusion) in rats. Figure 1A, B shows the total number of cocaine infusions and active lever presses under an FR2 reinforcement schedule, demonstrating that AM6527 (10 and 30 mg/kg, i.p.) failed to alter cocaine self-administration. One-way RM ANOVA over drug dose revealed no significant main effects of AM6527 treatment on the total number of cocaine infusions (*F*_2,12_ = 1.742; *p* = 0.217) and active lever presses (*F*_2,12_ = 1.017; *p* = 0.391).

Next, we examined the effects of AM6527 on cocaine-taking motivation under a progressive-ratio (PR) reinforcement protocol in rats. Figure 1C, D shows the total number of cocaine infusions and the breakpoint for cocaine SA, indicating that AM6527, at 10 and 30 mg/kg, significantly inhibited the breakpoint for cocaine SA under PR reinforcement. One-way RM ANOVA did not reveal significant main effects of AM6527 treatment on the total number of cocaine infusions (Fig. 1C, *F*_2,12_ = 3.31; *p* = 0.072), but revealed a significant treatment main effect on the breakpoint for cocaine SA (Fig. 1D, *F*_2,12_ = 7.60; *p* = 0.007). Post-hoc analyses revealed that AM6527, at 10 mg/kg (*p* < 0.01) and 30 mg/kg (*p* < 0.01), significantly reduced the breakpoint for cocaine SA. These findings suggest that AM6527 is effective in attenuating cocaine self-administration and motivation for cocaine seeking under PR reinforcement conditions.

### AM6527 reduces cocaine-induced reinstatement of drug seeking

Next, we examined whether AM6527 treatment affects cocaine-induced reinstatement of drug seeking. Indeed, AM6527, at 10 and 30 mg/kg, effectively attenuated reinstatement of cocaine-seeking behavior following extinction of cocaine self-administration (Fig. 1E). Two-way ANOVAs for the active lever responses revealed a significant AM6527 treatment main effect (F_2,21_ = 6.55, *p* < 0.01), session main effect (F_2,63_ = 54.81, *p* < 0.001), and treatment X session interaction (F_4,63_ = 4.30, *p* < 0.01). The same assays for the inactive lever responses didn’t reveal significant AM6527 treatment main effect (F_2,21_ = 1.93, *p* > 0.05), session main effect (F_2,63_ = 1.61, *p* > 0.05), and treatment X session interaction (F_4,63_ = 1.81, *p* > 0.05). Post-hoc analysis revealed a significant reduction in active lever responses after 10 mg/kg (*p* < *0.001*) or 30 mg/kg (*p* < *0.001*) AM6527 administration during the reinstatement session. There were no significant differences between treatment groups during the last session of self-administration or the last session of extinction (Fig. 1E). Overall, these findings suggest that AM6527 pretreatment can prevent reinstatement of cocaine-seeking behaviors.

### AM6527 reduces heroin self-administration under FR2 and PR reinforcement

**Fig. 2:**
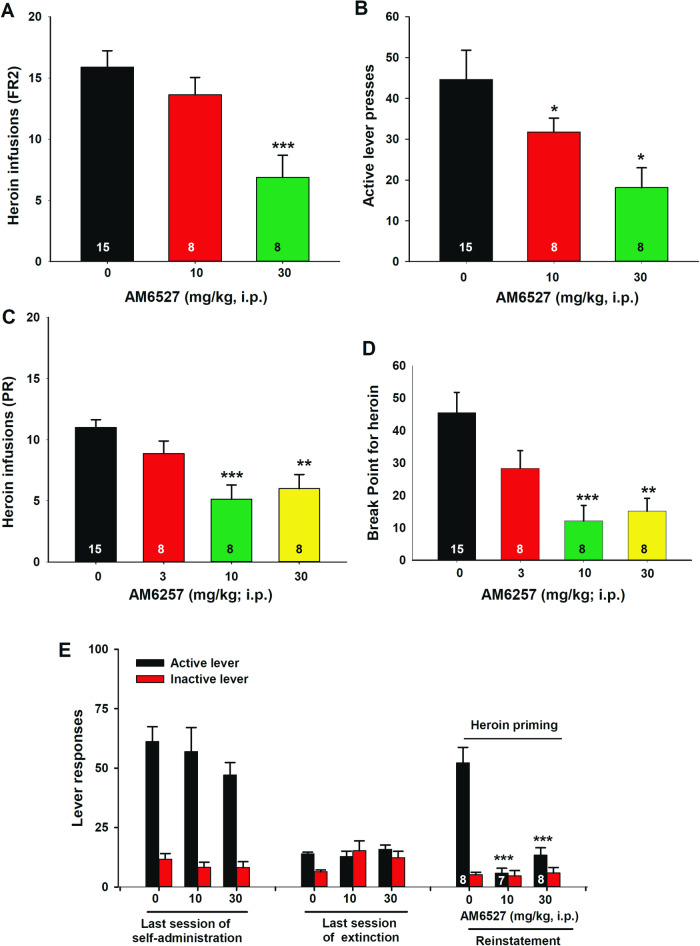
The effects of AM6527 on heroin self-administration and heroin-triggered reinstatement of heroin seeking in rats. AM6527 dose-dependently inhibited heroin self-administration assessed by the total number of infusions **A** or active lever presses **B** under FR2 schedule. AM6527 dose-dependently reduced the total number of heroin infusions **C** and breakpoint level **D** under PR schedule of reinforcement in rats. **E** Pretreatment with AM6527 inhibited heroin-induced reinstatement of drug-seeking behavior in rats. *p < 0.05, **p < 0.01; ***p < 0.001, compared to the vehicle control group.

Next, we evaluated the effects of AM6527 on heroin self-administration. We found that systemic administration of AM6527 significantly inhibited heroin self-administration under an FR2 reinforcement schedule, as assessed by the total number of heroin infusions (Fig. 2A) or active lever presses (Fig. 2B) within a 3-h session following acquisition of heroin self-administration. One-way ANOVA revealed a significant main effect of AM6527 treatment in the total number of heroin infusions (Fig. 2A, *F*_2,21_ = 9.22; *p* = 0.001) and active lever presses (Fig. 2B, *F*_*2,21*_ = 6.05; *p* < 0.01) without affecting the number of inactive lever presses (data not shown). Post-hoc analysis revealed a significant reduction in the total number of heroin infusions (*p* < 0.001) after 30 mg/kg AM6527 treatment and the number of active lever presses (*p* < 0.05 after 10 and 30 mg/kg AM6527 treatment).

We also examined the effects of AM6527 on motivation for heroin self-administration under PR reinforcement. We found that AM6527 was effective in reducing the total number of heroin infusions (Fig. 2C) and the breakpoint for heroin SA (Fig. 2D) under PR reinforcement. One-way ANOVA revealed a significant AM6527 treatment main effect on the total number of heroin infusions (Fig. 2C, *F*_3,35_ = 9.33, *p* < 0.001). Post-hoc analysis revealed significant reductions in the total number of heroin infusions following 10 mg/kg (*p* < 0.001) or 30 mg/kg (*p* < 0.01) AM6527 administration. Similarly, one-way ANOVA revealed a significant AM6527 treatment main effect on PR breakpoint (Fig. 2D, *F*_3,35_ = 7.71, *p* < 0.001). Post-hoc analysis revealed significant reductions in the breakpoint after 10 mg/kg (*p* < 0.001) or 30 mg/kg (*p* < 0.01) AM6527 administration.

### AM6527 reduces heroin-induced reinstatement of heroin seeking

Next, we examined whether AM6527 treatment blocks heroin-induced reinstatement of drug seeking. Figure 2E shows that AM6527, at both 10 and 30 mg/kg, robustly attenuated reinstatement of heroin seeking. Two-way ANOVAs for the active lever responses revealed a significant AM6527 treatment main effect (*F*_*2,20*_ = *11.27; p* < *0.001*), session main effect (F_2,60_ = 51.30, *p* < 0.001), and treatment X session interaction (F_4,60_ = 7.35, *p* < 0.001). The same assays for the inactive lever responses revealed a significant session main effect (F_2,60_ = 4.86, *p* < 0.05), but didn’t reveal a significant AM6527 treatment main effect (*F*_*2,20*_ = *0.37; p* > 0.05) and treatment X session interaction (F_4,60_ = 1.96, *p* > 0.05). Post-hoc analysis revealed a significant reduction in active lever presses after AM6527 treatment at 10 mg/kg (*p* < *0.001*) or 30 mg/kg (*p* < *0.001*) compared to the vehicle control group in the reinstatement session.

### Chronic administration of AM6527 inhibits heroin self-administration

**Fig. 3:**
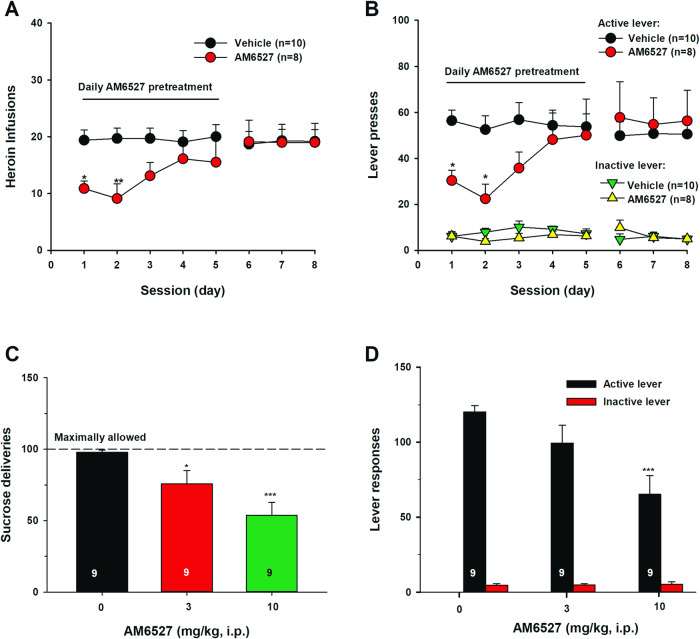
Effects of chronic AM6527 administration on heroin self-administration and a single injection of AM6527 on oral sucrose self-administration in rats. Chronic AM6527 administration (30 mg/kg, i.p., 30 min prior to test, for 5 consecutive days) inhibited heroin self-administration during the initial 2 days of tests as assessed by the number of heroin infusions **A** or the numbers of active and inactive lever responses under an FR2 reinforcement schedule **B**. A single injection of AM6527, at 3 and 10 mg/kg, dose-dependently inhibited oral sucrose self-administration as assessed by the number of sucrose deliveries **C** or the numbers of active and inactive lever responses **D** under an FR1 reinforcement schedule in rats. *p < 0.05; **p < 0.01, ***p < 0.001, com*p*ared to the vehicle control group.

We also examined the effects of chronic administration of AM6527 for 5 consecutive days on heroin self-administration under an FR2 reinforcement schedule in rats. Figure 3 shows the results, indicating that AM6527, at 30 mg/kg, produced a significant reduction in heroin self-administration only at the initial two days and then the effect was gone in the following 3 test days. Two-way RM ANOVA for the heroin infusion data (Fig. 3A) did not reveal a significant AM6527 treatment main effect (F_1,16_ = 1.77, *p* > 0.05), but revealed a significant time main effect (F_7,112_ = 3.61, *p* < 0.01) and treatment X time interaction (F_7,112_ = 4.59, *p* < 0.001). Post-hoc analyses revealed that pre-treatment with AM6527, at 30 mg/kg, significantly reduced the total number of heroin infusions on day 1 (*p* < 0.05) and day 2 (*p* < 0.01) of the 5 days of chronic treatment. A similar assay applied to the active lever-presses data (Fig. 3B) revealed a non-significant AM6527 treatment main effect (F_1,16_ = 0.71, *p* > 0.05), but a significant time main effect (F_7,112_ = 2.85, *p* < 0.01) and treatment X time interaction (F_7,112_ = 4.71, *p* < 0.001). Post-hoc analyses revealed that pre-treatment with AM6527, at 30 mg/kg, significantly reduced the total number of active lever-presses on day 1 (*p* < 0.05) and day 2 (*p* < 0.05) of the 5 days of chronic treatment. The total number of inactive lever-presses (Fig. 3B) were unaffected by AM6527 pre-treatment as a two-way RM ANOVA revealed no differences in treatment main effect (F_1,16_ = 0.24, *p* > 0.05), time main effect (F_7,112_ = 0.95, *p* > 0.05), and treatment X time interaction (F_7,112_ = 1.89, *p* > 0.05).

### AM6527 inhibits oral sucrose self-administration in rats

Previous studies have demonstrated that AM6527, at much lower doses (1, 2, 4, 8 mg/kg, i.p.) than those that inhibit heroin self-administration in the present study (3, 10, 20, 30 mg/kg, i.p.), dose-dependently inhibited oral food self-administration in rats [37]. In this study, we further assessed the effects of AM6527 on oral sucrose self-administration in rats to ascertain whether the varying effective doses of the drug were attributable to different reinforcers – drugs of abuse versus natural rewards. Our findings indicate that AM6527, administered at lower doses (3, 10 mg/kg, i.p.), indeed dose-dependently inhibits sucrose self-administration behavior (Fig. 3C, D). One-way repeated measures ANOVA revealed a significant treatment main effect on the total number of sucrose deliveries (Fig. 3C: F_2,24_ = 9.40, *p* < 0.001), active lever responses (Fig. 3D: F_2, 24_ = 1.89, *p* < 0.01), but not in inactive lever responses (Fig. 3D: F_2,24_ = 0.07, *p* > 0.05).

### AM6527 is not rewarding or aversive in electrical ICSS (eICSS) in rats

**Fig. 4:**
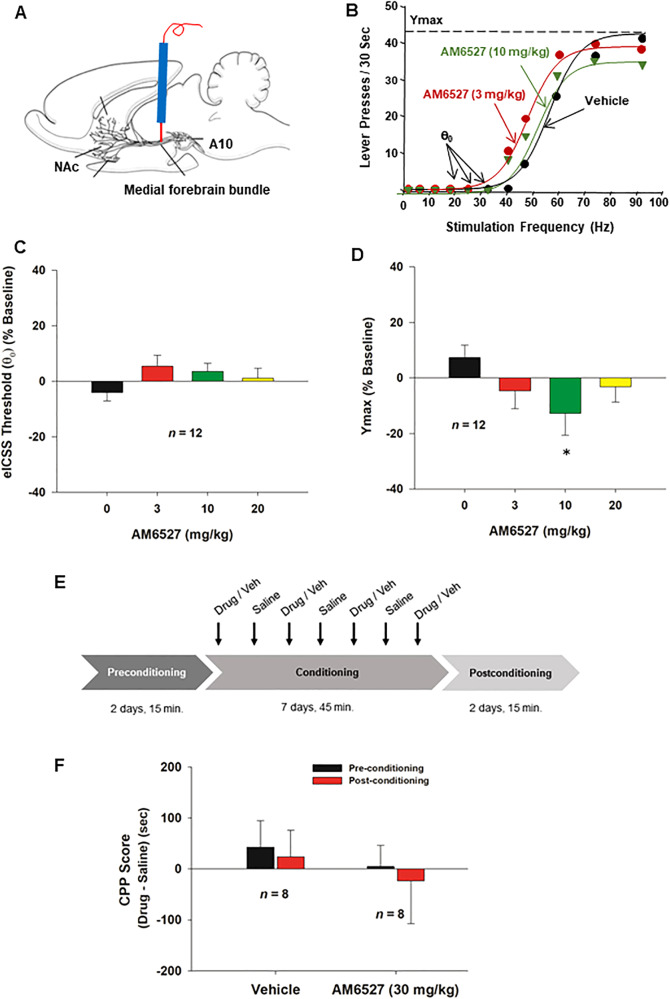
The effects of AM6527 on electrical intracranial self-stimulation (eICSS) in rats and conditioned place preference in mice. **A** A diagram showing the experimental methods for the eICSS m. **B** A diagram of stimulation-response curves showing the effects of AM6527 on eICSS. **C** Systemic administration of AM6527 did not alter the eICSS threshold **(**θ_0_) at any dose tested. **D** AM6527 significantly decreased the maximal operant responses (Ymax). **E** The timeline of the CPP experiment, where “Drug/Veh” represents AM6527 (30 mg/kg, i.p.) for one group (*n* = 8) and 5% cremophor for the other group (*n* = 9). **F** AM6527 did not produce CPP or CPA. *p < 0.05, compared to the vehicle control group.

To determine whether AM6527 by itself produces rimonabant-like aversive effects as we reported previously [32], we observed the effects of AM6527 on electrical ICSS threshold in rats (Fig. 4A). We found that electrical stimulation of the medial forebrain bundle produced robust ICSS behavior, which was not altered by AM6527. Figure 4B shows representative rate-frequency functions for ICSS, demonstrating ICSS threshold (θ_0_) and maximal operant responding (*Y*_*max*_). Systemic administration of AM6527 failed to alter the θ_0_ values (Fig. 4C). One-way RM ANOVA did not reveal AM6527 treatment main effects (Fig. 4C, *F*_3,33_ = 1.95, *p* > 0.05), suggesting that AM6527 is not rewarding or aversive. This is markedly different from the effect (e.g., a dose-dependent reduction in θ_0_ value) of rimonabant in our previous report [32]. Unexpectedly, systemic administration of AM6527 significantly decreased the Ymax (Fig. 4D, *F*_3,33_ = 3.27, *p* < 0.05) and post-hoc individual group comparisons revealed a significant reduction in Ymax after 10 mg/kg (*p* < 0.05), but not 20 mg/kg (*p* > 0.05), AM6527 administration.

### AM6527 did not produce CPP or CPA response in mice

Figure 4E shows the timeline for the CPP procedures. Each animal in the AM6527 group (*n* = 8) received 7 days of alternating AM6527 (30 mg/kg, i.p.) and saline (i.p.) injections during the conditioning phase. Each animal in the vehicle group received 7 days of alternating vehicle (5% cremophor, *n* = 8, i.p.) and saline (i.p.) injections during the conditioning phase. Figure 4F shows that AM6527, at the highest dose that reduced heroin self-administration and the breakpoint for cocaine-seeking, did not produce rewarding or aversive effects. Paired *t*-tests did not reveal a significant difference between the pre-conditioning and post-conditioning CPP scores of the AM6527 group (*p* > 0.05) or between the pre-conditioning and post-conditioning CPP scores of the vehicle group (*p* > 0.05).

### AM6527 reduces DA-dependent behavior in DAT-Cre mice

**Fig. 5:**
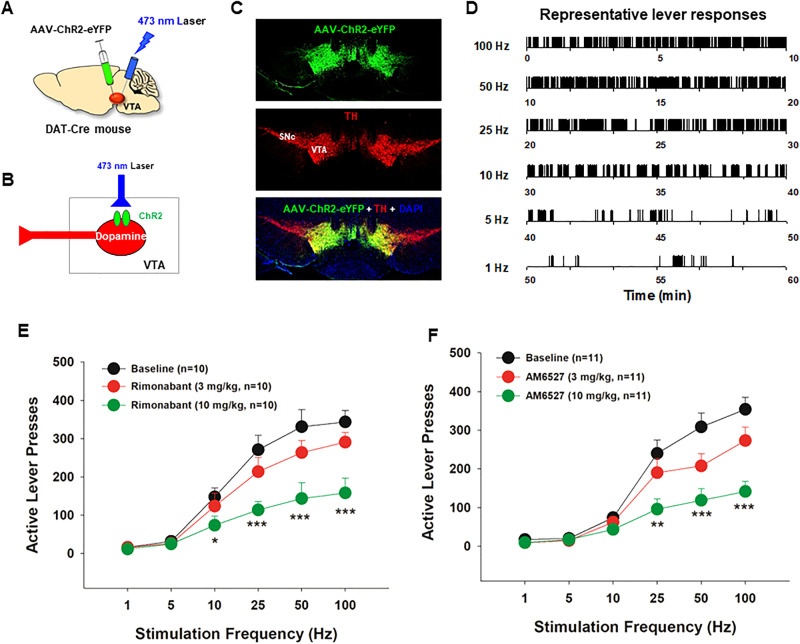
The effects of AM6527 on optical intracranial self-stimulation (oICSS) maintained by optogenetic stimulation of VTA DA neurons in DAT-Cre mice. **A, B** Schematics showing optical ICSS procedures. **C** Representative image showing AAV-ChR2-eYFP expression in VTA DA neurons. **D** Representative oICSS records illustrating stimulation frequency-dependent active lever responses. Systemic administration of rimonabant **E** or AM6527 **F** dose-dependently inhibited oICSS and shifted stimulation-lever response curve downward. *p < 0.05; **p < 0.01; ***p < 0.001, compared to the vehicle control group.

Lastly, we examined whether a DA-dependent mechanism underlies the AM6527’s action in drug-taking and drug-seeking behavior. Figure 5A shows the general experimental strategy, illustrating that Cre-dependent AAV-DIO-ChR2-eYFP vectors were injected unilaterally into the VTA to express light-sensitive ChR2 in DA neurons, and then optical fibers were implanted 1 mm above the VTA. We then trained mice to press lever to deliver laser stimulation into the VTA to activate DA neurons (Fig. 5B). Figure 5C shows representative images illustrating ChR2-eYFP expression in VTA tyrosine hydroxylase (TH)-positive DA neurons. Contingent laser delivery produced robust active lever responding (e.g., oICSS) in a stimulation frequency-dependent manner (Fig. 5D). Systemic administration of rimonabant or AM6527 dose-dependently inhibited oICSS (Fig. 5E, F). A two-way RM ANOVA revealed a significant rimonabant treatment main effect (Fig. 5E, *F*_*2,18*_ = 17.88, *p* < 0.001), stimulation frequency main effect (*F*_*5,45*_ = 41.53, *p* < 0.001), and treatment x frequency interaction (*F*_*10,90*_ = 7.63, *p* < 0.001). A similar assay also revealed a significant AM6527 treatment main effect (Fig. 5F, *F*_*2,20*_ = 7.96, *p* < 0.01), stimulation frequency main effect (*F*_*5,50*_ = 103.85, *p* < 0.001), and treatment x frequency main effect (*F*_*10,100*_ = 6.38, *p* < 0.001). These findings suggest that both rimonabant and AM6527 inhibit DA-dependent optical brain-stimulation reward.

## Discussion

In this study, we systemically evaluated the potential utility of the neutral CB1R antagonist AM6527 in treatment of cocaine and opioid use disorders. We found that AM6527 is effective in reducing cocaine self-administration under PR and heroin self-administration under both FR2 and PR reinforcement schedules, sucrose self-administration under FR1, as well as in blocking cocaine- or heroin-induced reinstatement of drug-seeking behavior. Unlike rimonabant, systemic administration of AM6527 neither altered electrical brain-stimulation reward in rats nor produced CPP or CPA in mice, suggesting that this neutral CB1R antagonist is not rewarding or aversive, and therefore, it may not produce rimonabant-like depression-like effects. In addition, both rimonabant and AM6527 dose-dependently inhibited DA-dependent brain-stimulation reward in oICSS. As cocaine or oxycodone (at low doses) potentiates oICSS as we reported previously [14, 47], the present finding in oICSS suggests that a DA-dependent mechanism may underlie the therapeutic effects of AM6527 against cocaine or opioid self-administration and reinstatement of drug-seeking behavior.

We note that AM6527 inhibited cocaine self-administration only under high-cost (PR) reinforcement, not under a low-cost (FR2) reinforcement schedule. This is consistent with our previous findings with other CB1R neutral antagonists AM4113 [32] and PIMSR [39], where none of these antagonists inhibit cocaine-self administration under low effort and high payoff (FR1, FR2) conditions. In contrast to cocaine, AM6527 appears to be more effective in attenuating heroin self-administration under both FR2 and PR reinforcement conditions, suggesting that this neutral CB1R antagonist is more suitable for the treatment of opioid use disorders. The mechanisms underlying the discrepancy in reducing cocaine versus heroin self-administration are unknown but may be due to differing mechanisms of cocaine or heroin action. Cocaine is a potent psychostimulant that blocks dopamine transporters in the nucleus accumbens (NAc), causing a rapid robust increase in NAc extracellular DA [49–51]. In contrast, heroin-enhanced DA is mediated indirectly via GABA-mediated disinhibition of VTA DA neurons [49], which displays a pattern of slow-onset, small amplitude, and long-term increase in DA [50, 51]. Thus, different dynamics in DA fluctuation may in part explain why CB1R antagonists are more effective in reducing opioid action than in reducing cocaine action. In addition, endocannabinoids modulate DA neuron activity in a similar manner to opioids, regulating glutamatergic and/or GABAergic inputs to DA neurons [52]. Thus, a possible interaction between opioids and endocannabinoids may occur in the mesolimbic DA system [45, 49, 53].

The precise mechanisms through which AM6527 attenuates the acute rewarding effects of cocaine or heroin remain unclear. It was reported that cocaine or heroin may increase endocannabinoid release in the VTA and NAc [54–59]. After releasing from post-synaptic neurons, such as VTA DA neurons [58], eCBs retrogradely diffuse back to activate presynaptic CB1Rs. This retrograde CB1R activation produces a reduction in GABA or glutamate release, which subsequently produces reward-enhancing effects via DA neuron modulation. Accordingly, CB1R antagonism at presynaptic terminals would block the eCB-mediated effects, producing anti-addictive effects [25].

It is important to remark that AM6527 may have higher translational potential than other CB1R neutral antagonists, such as AM4113 and PIMSR as AM6527 has significantly higher oral bioavailability, making it a more practical candidate for clinical trials [37]. Unexpectedly, chronic treatment with AM6527 failed to maintain an inhibitory effect during the 5 days of chronic treatment, suggesting possible development of tolerance to AM6527 after chronic drug administration. The underlying mechanisms is unclear. One possibility is that chronic AM6527 administration causes an adaptive change (for example, an increase) in CB1 receptor expression, which subsequently compromises the action of AM6527 on heroin self-administration. Another possibility is that chronic drug administration may cause an increase in enzymes in liver that metabolize the drug faster, causing a reduction in brain drug concentration. Thus, higher AM6527 doses may be required to maintain the therapeutic effects against heroin-taking behavior. Clearly, more studies are required to fully address this unexpected finding and explore an optimal chronic dose regimen for the use in future translational studies.

We note that significantly higher doses of AM6527 (10, 20, 30 mg/kg, i.p.) are required to inhibit cocaine and opioid self-administration compared to those (1, 2, 4, 8 mg/kg, i.p.) necessary for inhibiting food self-administration in rats [37]. We believe that the different effective doses of the drug could be due to the different reinforcers – drugs of abuse *versus* natural rewards, as lower doses (3, 10 mg/kg, i.p.) of AM6527 indeed effectively inhibited oral sucrose self-administration. In essence, AM6527 demonstrates greater efficacy in attenuating natural (food) rewards compared to highly active drugs.

In this study, we also evaluated possible side-effects of AM6527 itself in eICSS and CPP tests, two commonly used models to evaluate a drug’s abuse potential or aversive effects[25]. We and others previously reported that high doses of rimonabant inhibits electrical ICSS [32, 60], but failed to produce CPP or CPA (see a comprehensive review by [25]). In the present study, we found that AM6527 failed to alter eICSS threshold and also failed to produce CPP or CPA, suggesting that AM6527 may have no aversive side-effects. We note that AM6527, at an intermediate dose (10 mg/kg), significantly lowers Ymax in the eICSS. The reasons underlying this reduction in Ymax are unknown. As a higher dose (20 mg/kg) did not alter the ICSS threshold (θ_0_) or Ymax, it is suggested that this mild locomotor effect may not be a serious concern in developing this CB1R antagonist for the treatment of SUDs.

Interestingly, both rimonabant and AM6527 produced a significant reduction in DA-dependent oICSS in a dose-dependent manor. The reasons underlying these disparate findings in eICSS and oICSS with AM6527 are unclear. One possibility is that electrical pulses into the brain may nonspecifically stimulate multiple types of neurons or nerve fibers in the medial forebrain bundle, while optical stimulation selectively activates midbrain DA neurons without altering any other phenotypes of neurons. Thus, it is likely that different neural substrates may underlie the action of AM6527 in eICSS *versus* DA-dependent oICSS. For example, it was reported that a serotonin, not DA or norepinephrine, -related mechanism may underlie rimonabant-induced depressant-like effects [61]. As serotoninergic fibers in the middle forebrain bundle may also be activated in eICSS, but not in oICSS, this may in part explain why AM6527 produces different effects in eICSS and oICSS. Given that rimonabant and both the neutral antagonists PIMSR and AM6527 all inhibit DA-mediated oICSS as shown in this report and previous report [39], it is suggested that DA-dependent mechanisms may underlie their therapeutic anti-addictive effects, while a non-DA mechanism may underlie the depressive and aversive effects of the inverse agonizts such as rimonabant and AM251 [25, 61].

In conclusion, our study contributes to the growing evidence demonstrating CB1R neutral antagonists as therapeutic candidates for treating SUDs. The present study shows that the CB1R neutral antagonist AM6527 inhibits cocaine and heroin self-administration as well as reinstatement of drug-seeking behavior, while itself is not rewarding or aversive, suggesting that AM6527 and other neutral CB1R antagonists are superior to rimonabant as new pharmacotherapies for treating cocaine and opioid use disorders.

## Data Availability

All data are presented in the main manuscript.
